# Early intervention program reduces stress in parents of preterms during childhood, a randomized controlled trial

**DOI:** 10.1186/1745-6215-15-387

**Published:** 2014-10-04

**Authors:** Inger Pauline Landsem, Bjørn Helge Handegård, Jorunn Tunby, Stein Erik Ulvund, John A Rønning

**Affiliations:** Child and Adolescent Department, University Hospital of Northern Norway, Tromsø, Norway; UiT, Health Faculty, The Arctic University of Norway, Tromsø, Norway; UiT, RKBU Nord, The Arctic University of Norway, Tromsø, Norway; Department of education, University of Oslo, Oslo, Norway

**Keywords:** Early intervention, Preterm, Parenting stress, Longitudinal study, Long-term follow-up

## Abstract

**Background:**

It is well documented that heightened levels of parenting stress have a negative influence on children’s socio-emotional and behavioral development. Parenting stress may therefore be regarded as an outcome variable in its own right. This study investigated whether a sensitizing intervention influences stress reported by parents of prematurely born children until the children were age nine.

**Methods:**

Preterm infants (N =146, birth weight <2,000 g) were randomized to intervention (N =72) with the Mother-Infant Transaction Program (MITP) or a preterm control group (N =74) that received standard hospital care. A term reference group comprised 75 healthy, full-term neonates. Parents reported on the Parenting Stress Index (PSI) when the children were 6 months, 1, 2, 3, 5, 7 years old and on the PSI-Short Form (PSI-SF) at age 9. Main outcomes were the mother’s and father’s reports of total, child and parent-related stress. Cross-sectional and longitudinal analyses were performed using linear mixed models (LMM), taking dependency in the data caused by twin pairs and repeated measures into account. Response rates were high across all follow-ups, and still reached 85% from mothers and 72% from fathers at 9 years.

**Results:**

Mothers in the intervention group reported better longitudinal development of child-related stress than mothers of preterm controls, as they perceived their children as being more adaptable and less moody throughout childhood until the age of seven. Less stress in the intervention group was revealed by cross-sectional analysis of maternal reports at all ages, while fathers reported similar differences at ages three and five. Parents in the intervention group reported stronger agreement on several stress scores on several occasions. Fathers with high interventional participation (mean 54%) reported significantly less stress at age nine than those who participated less. Both parents in the intervention group reported levels of stress similar to those experienced by the term reference group at all follow-ups, while differences between the preterm control and term reference groups increased.

**Conclusions:**

This early intervention reduces stress among parents of prematurely born children to a level reported by parents of term-born children and enhances agreement between parents.

**Trial registration:**

Clinical Trials Gov identifier NCT00222456, 05.09.2005.

## Background

High levels of parental stress have frequently been reported when children are born preterm [1, 2]. Prematurely born children are at increased risk of behavioral problems compared to term-born infants [3, 4]. Reducing the levels of stress is important not only for improving parental psychological health but also because it may improve the efficacy of interventions that target these children’s behavioral problems [5]. These interventions thus justify the assessment of parenting stress as an important outcome in the evaluation of an early intervention program [4, 5].

Parenting stress has been defined as a mismatch between perceived resources, expectations and actual caregiving demands [5], and covers stress from different origins that places the parent-child relationship under lasting pressure [6, 7]. Schappin *et al*. [8] concluded that stress experienced by parents of preterm infants has gradually decreased over the last thirty years, probably due to increased quality of care for preterm infants. On the other hand, Treyvaud *et al*. [9] recently reported that parents of very preterm children continue to report more child- and parent-related stress lasting until children’s age of (hereinafter ‘age’) seven. This may indicate that parents of prematurely born children find it just as difficult to interpret and adapt to the immature expressions of a preterm-born infant today as they did 30 years ago, irrespective of their child’s medical condition. The gap between normal parental expectations and infant expressive capacity needs to be reduced following the birth of a preterm child.

Abidin has described stress as a multidimensional concept; cumulative, highly influenced by the environment, and a result of transactions between parent and child that promote negative feelings in the parent [6]. Based on this model, the Parenting Stress Index-Full Form (PSI-FF) was created to capture (a) stress related to the parent’s personality and vulnerability; (b) child characteristics as perceived by the adult; (c) life events and; (d) the extent of supportive environment that parents experience. The PSI-FF distinguishes between different aspects of perceived stress in child and parental dimensions, and the Child Domain in particular reveals parental perceptions of stress related to children’s individual characteristics.

Several studies have reported that high levels of parenting stress may disrupt parental sensitivity and responsiveness and lead to ineffective, dysfunctional parenting with possible negative impacts on child development [5, 9–11]. A meta-analysis concluded that significantly more child-related stress was reported by parents of prematurely-born children than those of term-born, in areas such as distractibility/hyperactivity, demandingness and acceptability among children at ages between 1 month and 12 years [8]. These results are in accordance with studies that have reported prematurely-born children to be more demanding than term-born because of immature expression; poor self-regulation and restricted capacity to interact socially in environments that are noisy, bright or are generally characterized by non-optimal stimuli [12].

A premature birth may also disturb the maturation of parental attachment bonds, which are regarded as an essential part of the parental behavioral system, preparing adults for caregiving [13–15]. Parental bonding is supposed to have a special impact on parents’ capacities to cope with stress, as significant associations have been reported between low levels of stress and parental reports of a preferred parental bonding type (high level of care and low level of control) at age seven [16]. Parental attachment bonds may be regarded as complementary to the infants’ care-seeking attachment and deal with emotional ties that involve the development of feelings of love [15]. Parental attachment is in line with Abidin’s construction of an Attachment subscale in the PSI-FF, which is loaded with questions that address parents’ perceived difficulties in establishing an emotional closeness to the infant [6, 15]. Prematurity has been found to be a strong predictor of diminished caregiving quality, while research has reported a weak impact of prematurity on the development of child attachment [13, 17]. All aspects mentioned above underline the importance of strengthening parents’ ability to cope with the delivery of a preterm child and to manage this stressful situation.

Several interventions that aim to ameliorate these problems have been investigated. Key components of interventions, all of which involve efforts to improve parental outcomes and subsequently child outcomes, have been described as psychosocial support, parent education and therapeutic developmental interventions targeting the infant [18]. The meta-analysis by Bakermans-Kranenburg, van Ijzendoorn *et al*. concluded that interventions that were able to enhance parental sensitivity were the most effective [19]. This study evaluates whether a modified version of the Mother-Infant Transaction Program (MITP) [4] could strengthen parents’ perceptions of their preterm child and prevent the increased levels of parenting stress that have repeatedly been reported [20, 21]. The MITP was designed to facilitate social availability and interactions with the newborn infant and thereby strengthen parental enthusiasm, pleasure and empowerment [4]. Our group has previously reported lower levels of parenting stress in the intervention group until age two [22, 23]. Moreover, the intervention appears to improve the children’s socio-emotional and behavioral development [24, 25]. On the basis of these findings, we hypothesized that preterm intervention (PI) parents would continue to report less stress throughout childhood, as stability in parents’ perception of parenting stress is well documented [20, 21]. The following questions were addressed: 1) has the early intervention influenced the longitudinal development of parenting stress as reported by mothers and fathers? 2) are there cross-sectional differences between the preterm groups in mothers’ and fathers’ reports of parenting stress at any age until nine, when controlled for repeated measures? 3) how is the development of stress reported by parents in the two preterm groups compared with that reported by parents of term controls?

## Methods

### Participants

This study is a part of the Tromsø Intervention Study on Preterms (TISP); a randomized, controlled study of preterm infants with birth weight (BW) <2000 g, recruited between March 1999 and September 2002 (Rønning, Ulvund, Dahl & Kaaresen, 1998, unpublished research protocol). Preterm infants were randomized into blocks of six by using computer-generated numbers, to form an intervention group (PI, N =72) and a preterm control group (PC, N =74), and stratified according to gestational age (GA) <28 and GA ≥28 weeks. Healthy newborns (GA ≥37 weeks) were also recruited from the neonatal nursery to form a term reference group (TR, N =75). Written, informed consent was received from all participants before inclusion. Preterm controls (PC) followed the neonatal intensive care unit (NICU) guidelines for discharge of preterm infants, while term controls (TR) were routinely examined once by a pediatrician on their third day of life. Baseline data for each study group have previously been described in detail elsewhere [22, 23], and are shown in Table 1.

**Table 1:** **Birth, medical and demographic information**

	PI group	PC group	TR group
	N =72	N =74	N =75
Infant characteristics	1,396 ± 429	1,381 ± 436	3,619 ± 490
BW, mean ± SD, g	20 (28)	20 (27)	
400 to 1000 g, n (%)	15 (21)	20 (27)	
1001 to 1500 g, n (%)	37 (51)	34 (46)	
1501 to 2000 g, n (%)	30.2 ± 3.1	29.9 ± 3.5	39.3 ± 1.3
GA, mean ± SD, week	17 (24)	19 (27)	
<28 week, n (%)	36 (50)	19 (27)	
28 to 32 week, n (%)	38 (53)	37 (50)	
≥33 week, n (%)	16 (22)	18 (24)	
Boy, n (%)	38 (53)	39 (53)	40 (54)
Twin, n (%)	16 (22)	14 (19)	0
Prenatal steroid use, n (%)	53 (74)	57 (77)	
SNAP II, mean ± SD	8.3 ± 10.9	10.4 ± 11.3	
CRIB score, mean ± SD, N =85	3.2 ± 2.8	2.7 ± 2.9	
Received ventilation, n (%)	29 (40)	37 (50)	
Duration of ventilation, n (%)	7.0 ± 18.6	7.1 ± 17.3	
Postnatal steroid use, n (%)	9 (13)	10 (14)	
Oxygen therapy at 38 week GA, n (%)	11 (15)	14 (19)	
Abnormal cerebral ultrasound, n (%)			
IVH grade 1 or 2	7 (10)	8 (11)	
IVH grade 3 or 4	3 (4)	5 (7)	
Periventricular leukomalacia	4 (6)	8 (11)	
Maternal and social characteristics			
Mother’s age, mean ± SD, years	30.8 ± 6.1	29.1 ± 6.4	29.7 ± 6.1
First-born child, n (%)	40 (56)	37 (54)	27 (37)
Mother’s education, mean ± SD, years, N =131	14.6 ± 2.8	13.5 ± 3.2	14.9 ± 2.8
Father’s education, mean ± SD, years, N =131	13.8 ± 3.1	13.5 ± 3.2	14.4 ± 3.2
Mother’s monthly income, mean ± SD, 1,000 Norwegian kroner, N =131	15.8 ± 7.7	14.6 ± 6.7	15.9 ± 8.0
Father’s monthly income, mean ± SD, 1,000 Norwegian kroner, N =131	21.1 ± 8.7	19.9 ± 8.1	21.9 ± 9.8

*Abbreviations:* *BW* birth weight, *CRIB* Clinical Risk Index for Babies, *GA* gestational age, *IVH* intraventricular hemorrhage, *PC* preterm control group, *PI* preterm intervention group, *SNAP* Score for Neonatal Acute Physiology, *TR* term reference group.

### Intervention

The intervention program was a modified version of the MITP [4] aimed at 1) enhancing parents’ understanding of their child’s expressions, and 2) promoting a sensitive, positive and practical transaction between parents and child. Eight nurses were trained to perform the intervention and each family was guided by the same nurse during all the sessions. Each intervention consisted of 7 hour-long sessions with parents and their baby during the last week before discharge, and 4 home visits at 1, 2, 4, and 12 weeks post-discharge [4]. The modification of the MITP included an initial session during which parents could vent their feelings about their preterm child. Mothers participated in all sessions while the fathers’ mean participation rate was 6.5 sessions (SD =3.4), which constituted 54% of the intervention program. In the first session the parents and the intervention nurse investigated the child’s capacities, focusing on the baby’s readiness and social communication abilities. During the following sessions, the parents were helped to recognize and be sensitive to behavioral cues, signs of disturbed regulation and stress in the child’s physiological, motor and state organization. The guidance was given while they observed the infant together, and all comments, questions and suggestions from the parents were appreciated. Finally, this understanding was applied to daily care by helping parents to make adjustments to their child’s strengths and vulnerabilities, in order to reduce stress levels and maximize the parents’ social engagement with their babies. During the four home visits, these topics were revisited and fine-tuned to individual needs, especially in connection with the child’s temperament, which was one of the main topics of the third home visit. The families had no other contact with the intervention nurses. All sessions were documented by logbooks written by the interventionists, and implementation according to the intervention manual [4] was ensured by logbook reviews carried out by the study director (JAR).

### Measures

At the ages of 6 months, 1, 2, 3, 5 and 7 years, parents completed the Parenting Stress Index-Full Form (PSI-FF, third edition) while the Parenting Stress Index-Short Form (PSI-SF) was used when the children were 9 years old [6]. The PSI-FF consists of 120 questions covering three main dimensions of stress (child, parent and life stress) while the PSI-SF consists of 36 questions extracted from the parent- and child-related dimensions. A five-point Likert scale ranging from ‘strongly agree’ to ‘strongly disagree’ made up the response alternatives on both questionnaires. At 6 months, only one parent reported (mostly mothers) while mothers and fathers reported separately on all the subsequent occasions.

The PSI-FF consists of two main dimensions: Child Domain (47 items covering the subscales: Distractibility, Adaptability, Reinforces Parent, Demandingness, Mood and Acceptability), and Parent Domain (54 items covering the subscales: Perceived Competence, Isolation, Attachment, Health, Role Restriction, Depression and Relation to Spouse). A Total Stress (TS) score was also computed on the basis of all items except the life-stress questions. The PSI-SF is reported as a Total Stress score and by three subscales, each of which consists of 12 items: Parental Distress (PD), Parent-Child Dysfunctional Interaction (P-CDI) and Difficult Child (DC). Some questions in both questionnaires are used to calculate a Defensive Responding score, which indicates the degree of possible inconsistent/denial reporting from respondents.

Both PSI-FF and PSI-SF are frequently used in research [26, 27], and the correlation between Total Stress scores on these two measures is described as high (0.87) [6, 28]. The PSI-SF, DC subscale consists solely of items from the Child Domain in PSI-FF, and the Parental Distress subscale items from the Parent Domain. The P-CDI subscale includes items from both the Child and Parent Domains and focuses on the parental perception of transactions with their child and their expectations about the child’s behavior [6]. The Norwegian versions of both PSI-FF and PSI-SF were translated by Rønning and Abidin, and were used in this study with the permission of Abidin and Psychological Assessment Resources, Inc. (PAR). The questionnaires have some literal differences, in that questions in the PSI-SF may be perceived as more negative and definitive than those in the original PSI-FF format. The Life Stress (LS) questionnaire is part of the PSI-FF and was also used at age nine. The LS questionnaire consists of 22 items covering major life events in the family that are assumed to be challenging, even though they not are directly associated with child or parental challenges.

### Follow-up procedures

All the participating children received the same medical, developmental, and psychosocial assessments with recommendations about contacting other services if needed (age 6 months, 1, 2, 3, 5, 7, 9 years). Questionnaires were sent to the families approximately two weeks before each assessment. TISP was approved by the Regional Committee for Medical Ethics (2010/2153/REK nord) and the Norwegian Data Inspectorate on three occasions (in 1999, 2005, and 2010).

### Analysis

**Figure 1:**
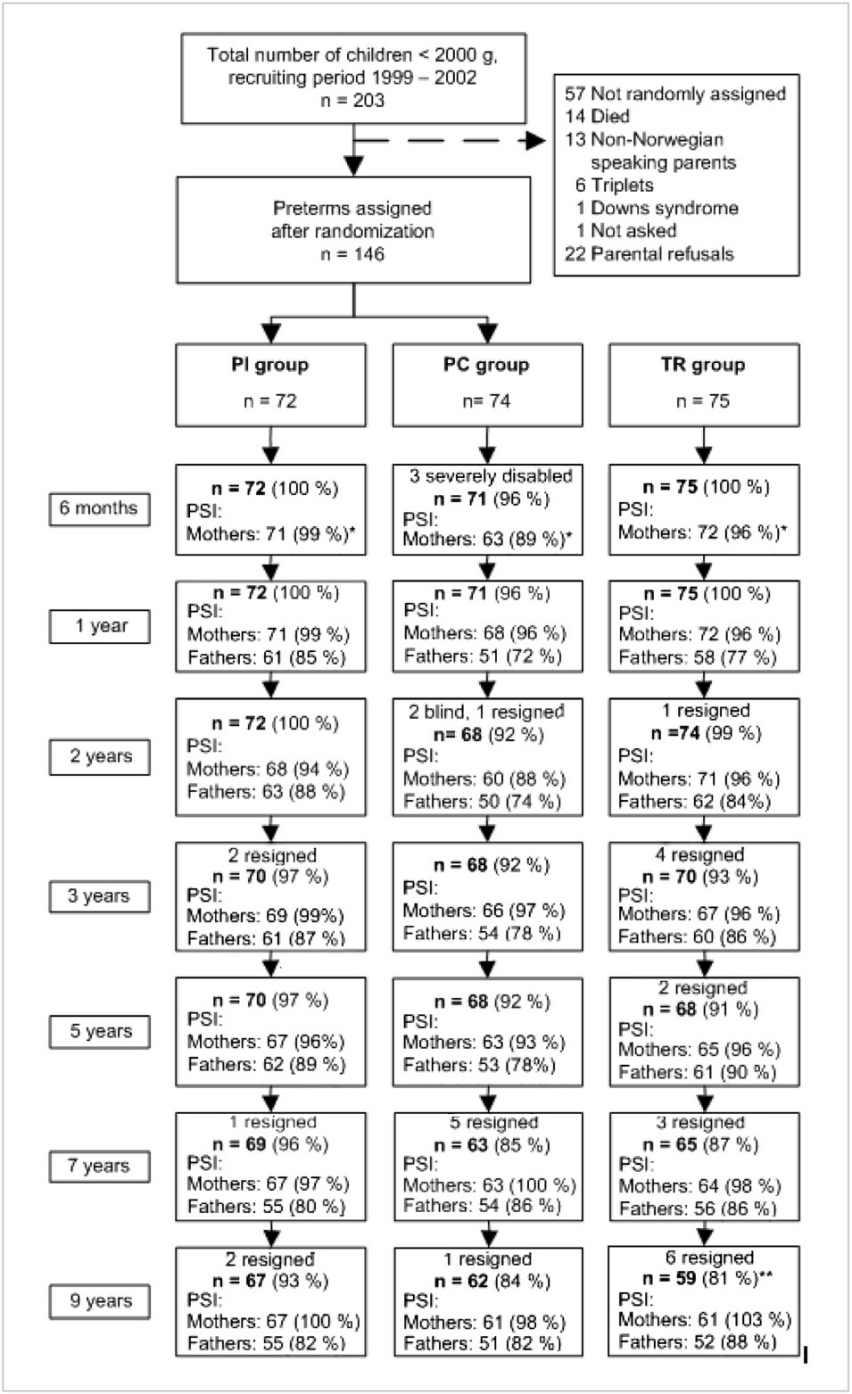
**Study flow and parents’ reports on Parenting Stress Index (PSI) from 6 months to 9 years of age.** At age 9, two mothers in the TR group reported on PSI but their child did not attend the follow-up session.

Because of repeated measures and the clustering effects of twin pairs, all longitudinal and cross-sectional analyses were performed by multilevel modeling (linear mixed models (LMM), SPSS statistics, version 20, SPSS Inc., Chicago, IL, USA). In the longitudinal analysis, time was treated as a continuous variable. In the cross-sectional analysis, predicted mean group differences with 95% confidence intervals (CI) were calculated. These analyses were also based on a longitudinal model, but in these cases time was treated as a categorical variable [29]. By varying the reference time point in the analysis, predicted group differences could be calculated. To assess agreement between parents, intraclass correlations (ICC) were computed, and the difference between the two independent intraclass correlation coefficients for the PI and the PC groups was tested as described by Alsawalmeh and Feldt [30]. The impact of variable intervention participation by fathers was analyzed by LMM and adjusted to take into account the clustering effects of twin pairs, and effect sizes in this case was given by Pearson correlations. Effect sizes (ES) created by the use of Hedges’ g are reported on predicted cross-sectional differences in mean scores between the PI and PC groups [31]. A *P*-value < .05 was considered significant. Randomization and inclusion criteria resulted in well-balanced study groups with one exception. Mothers in the PI group had an average of one more year of education at inclusion time (Table 1). The response rates were good throughout the study, still reaching 85% among mothers and 72% among fathers across all groups at age 9 (Figure 1).

## Results

### Longitudinal development of parenting stress in the PI and PC groups

**Figure 2:**
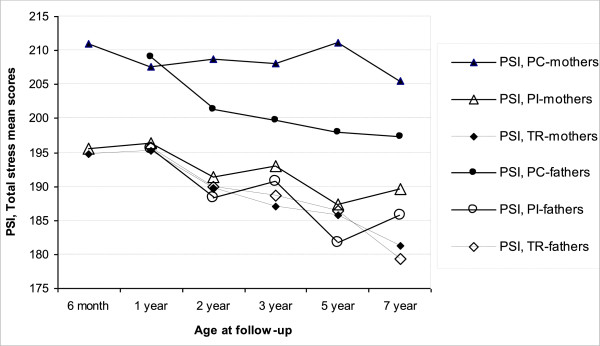
**Parenting Stress Index (PSI), Total Stress reported by mothers and fathers in the preterm intervention (PI), preterm control (PC) and term control (TR) groups from 6 months until 7 years.**

No group by age interactions were uncovered on PSI, TS as reported by mothers or fathers from age 6 months until 7 years (Figure 2). Mean scores in all three groups were low compared to the American normative mean score (222 points) reported by Abidin [6].

**Figure 3:**
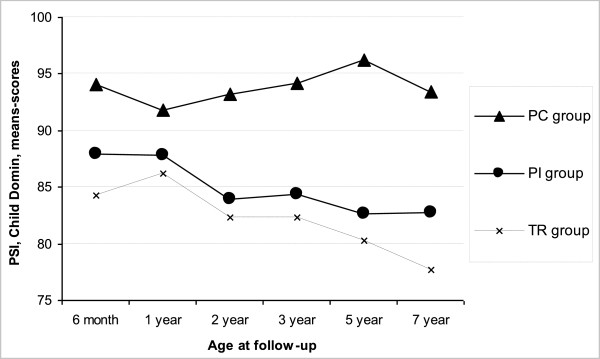
**Mothers’ reports on Parenting Stress Index (PSI)-Child Domain from age 6 months until 7 years.**

In PSI-Child Domain a group by age interaction was reported by mothers from age 6 months until 7 years (F(5,642) =2.7, *P* = .02). While PI mothers reported child-related stress as being at its highest at 6 months and decreasing until age 7, PC mothers reported increasing levels from age one until 5 years (Figure 3).

The interaction concerning child-related stress in maternal reports may primarily be a result of two similar interactions in the subscales Adaptability (F(5,654) =3.3, *P* = .006) and Mood (F(5,663) =3.2, *P* = .007). All group by age interactions continued to be significant when controlled for maternal education (Table 1). No group by age interactions were reported either by mothers in PSI-Parent Domain or by fathers in either child- or parent-related stress.

### Parenting stress in the PI and PC groups at different ages

Cross-sectional differences (at age 6 months, 1, 2, 3, 5, 7 and 9 years) in parental reports of child- and parent-related stress are reported first (stress reported at age 6 months, 1 and 2 years has been reported earlier [22, 23], but now predictions are based on a longitudinal model). Next, significant differences in different aspects of parenting stress (PSI, subscales) are reported. Lastly, agreement between parents in the PI and the PC group are compared.

Mother’s reports of child, parent and total stress are displayed in Table 2. Differences between preterm groups were mostly around 0.5 SD, and ESs were at their highest at 5 years in CD (ES =0.62). Mothers in the PI group reported less total stress than mothers in the PC group at every follow-up from age one until nine. Similarly, they reported significantly less child-related stress from age two until nine and less parent-related stress at ages two, three and five.

**Table 2:** **Mean scores and predicted mean differences in Parenting Stress Index (PSI) main dimensions as reported by mothers in the preterm intervention (PI) and preterm control (PC) groups**

		***N*** ^a^ PC, PI	PC group mean (SD)	PI group mean (SD	Predicted mean difference, (95% CI) ^c^	***P***	ES ^b^	TR group mean (SD)
6 months	Child Domain	68,72	94.3 (15.4)	88.1 (14.6)	5.1 (-1.0,11..2)	.1	0.34	84.3 (13.3)
	Parent Domain		116.9 (20.8)	108.9 (19.3)	5.3 (-2.2,12.7)	.2	0.26	110.4 (20.3)
	Total Stress		211.6 (34.3)	195.8 (30.2)	9.6 (-2.9,22.1)	.1	0.30	194.8 (30.6)
1 year	Child Domain	68,71	92.3 (14.6)	87.6 (17.8)	4.3 (-1.8,10.4)	.2	0.26	86.2 (15.4)
	Parent Domain		116.7 (20.3)	107.9 (20.6)	7.9 (0.5,15.3)	.04	0.39	110.1 (20.5)
	Total Stress		208.9 (32.6)	195.5 (35.5)	12.9 (0.5,25.2)	.04	0.39	195.3 (33.0)
2 years	Child Domain	60,68	93.5 (16.3)	84.2 (16.1)	9.8 (3.5,16.0)	.002	0.60	82.3 (15.2)
	Parent Domain		116.2 (18.9)	107.1 (19.6)	9.1 (1.6,16.6)	.04	0.47	107.2 (21.7)
	Total Stress		210.0 (30.8)	191.6 (33.1)	19.1 (6.5,31.6)	.003	0.60	189.7 (34.8)
3 years	Child Domain	66,69	95.3 (19.7)	84.2 (16.3)	10.5 (4.4,16.7)	.001	0.58	82.3 (14.9)
	Parent Domain		115.3 (21.4)	107.2 (20.6)	8.1 (0.6,15.6)	.04	0.39	105.0 (18.0)
	Total Stress		210.6 (37.8)	191.7 (33.6)	18.4 (6.0,30.7)	.004	0.52	187.1 (31.1)
5 years	Child Domain	63,67	97.1 (22.1)	82.3 (19.2)	12.9 (6.8,19.1)	< .0005	0.62	80.3 (15.1)
	Parent Domain		115.9 (23.4)	104.8 (21.8)	8.4 (0.9,15.9)	.03	0.37	105.9 (21.7)
	Total Stress		213.0 (39.9)	186.8 (37.9)	21.8 (9.5,34.2)	.001	0.56	185.8 (33.8)
7 years	Child Domain	63,67	94.0 (21.0)	82.7 (19.1)	9.7 (3.6,15.9)	.002	0.48	77.7 (15.2)
	Parent Domain		113.9 (23.2)	105.8 (22.8)	5.7 (-1.9,13.3)	.1	0.25	103.7 (24.3)
	Total Stress		207.9 (39.8)	188.8 (38.6)	15.8 (3.4,28.2)	.01	0.40	181.2 (37.5)
9 years	Difficult Child	61,67	25.4 (9.1)	21.3 (8.9)	3.9 (1.6,6.1)	.001	0.43	19.1 (5.4)
	Parental Stress		21.4 (7.1)	19.0 (6.0)	2.1 (-0.1,4.3)	.06	0.32	19.1 (6.5)
	Parent-Child Difficult Interaction		20.3 (5.7)	17.6 (5.6)	2.4 (0.6,4.3)	.01	0.42	16.4 (4.4)
	Total Stress		67.0 (19.6)	57.9 (17.9)	8.3 (3.0,13.6)	.002	0.44	54.7 (14.9)

^a^Number of reports from mothers in the PC and PI groups.

^b^Effect size, Hedges’ g, based on predicted mean differences.

^c^Analyzed with linear mixed models (LMM), adjusted for repeated measures and clustering effects of twin pairs.

ES, effect size.

Fathers in the PI group reported significantly less child-related stress (PSI, CD) than fathers in the PC group at 2, 3 and 5 years and less total stress at age 5 (Table 3). Significant correlations were uncovered between reported stress and the number of interventions in which PI fathers had participated. Fathers who had participated less reported more stress at age 3 in: Total Stress (t(57) =2.2, *P* = .03, r = -0.32) and child-related stress (t(58) =3.0, *P* = .004, r = -0.37). A similar result was found at age 9; Total Stress (t(58) =2.5, *P* = .02, r = -0.33); Parent-Child Difficult Interaction (P-CDI) (t(52) =3.0, *P* = .01, r = -0.38) and DC (t(50) =2.44, *P* = .02, r = -0.32).

**Table 3:** **Mean scores and adjusted mean differences on Parenting Stress Index (PSI), main dimensions as reported by fathers in the preterm intervention (PI) and preterm control (PC) group**

	Fathers reports	***N*** ^a^ PC, PI	PC group mean (SD)	PI group mean (SD	Predicted mean difference, (95% CI) ^c^	***P***	ES ^b^	TR group mean (SD)
1 year	Child Domain	51,61	96.0 (13.9)	89.3 (15.8)	4.5 (-1.8,10.8)	.2	0.30	89.3 (12.2)
	Parent Domain		113.5 (21.2)	105.3 (19.6)	6.6 (-1.4,14.6)	.1	0.33	106.3 (15.3)
	Total Stress		209.4 (32.7)	194.6 (33.9)	10.3 (-3.1,23.6)	.1	0.31	195.7 (24.9)
2 years	Child Domain	50,63	92.6 (13.1)	86.2 (16.9)	6.6 (0.4,12.9)	.04	0.43	85.8 (11.3)
	Parent Domain		108.4 (17.8)	103.3 (24.6)	5.6 (-2.3,13.6)	.2	0.26	103.8 (16.9)
	Total Stress		200.4 (27.2)	189.4 (40.3)	11.7 (-1.6,25.2)	.08	0.33	190.0 (24.9)
3 years	Child Domain	54,61	93.8 (14.8)	86.2 (16.6)	7.6 (1.3,13.8)	.02	0.48	85.9 (12.7)
	Parent Domain		105.4 (18.2)	103.7 (23.8)	3.0 (-4.9,11.0)	.5	0.14	102.6 (17.0)
	Total Stress		199.9 (31.0)	189.3 (37.5)	10.4 (-3.0,23.7)	.7	0.30	188.7 (27.4)
5 years	Child Domain	53,62	93.9 (20.2)	82.1 (15.6)	9.8 (3.6,16.1)	.002	0.55	85.1 (15.5)
	Parent Domain		105.2 (20.7)	98.1 (21.8)	5.3 (-2.7,13.3)	.2	0.25	101.4 (18.8)
	Total Stress		199.7 (37.3)	180.4 (34.9)	14.6 (1.3,28.0)	.03	0.41	186.4 (32.1)
7 years	Child Domain	54,55	92.4 (19.9)	87.2 (19.3)	4.7 (-1.6,11.0)	.1	0.24	80.8 (16.2)
	Parent Domain		105.2 (21.9)	100.2 (24.6)	4.2 (-3.8,12.2)	.3	0.18	98.7 (18.5)
	Total Stress		197.6 (38.5)	186.4 (40.5)	9.0 (-4.4,22.4)	.2	0.23	179.4 (32.6)
9 years	Difficult Child	51,55	21.6 (7.9)	21.3 (8.7)	0.6 (-1.6,2.7)	.6	0.07	18.8 (5.9)
	Parental Stress		19.5 (6.4)	20.5 (8.6)	-0.1 (-2.5,2.3)	.9	0.01	18.3 (5.6)
	Parent -Child Difficult Interaction		19.0 (5.6)	18.6 (6.0)	0.5 (-1.5,2.5)	.6	0.09	16.4 (4.5)
	Total Stress		60.1 (17.6)	60.3 (21.3)	1.0 (-4.7,6.7)	.7	0.05	53.5 (14.9)

^a^Number of reports from mothers in the PC and PI group.

^b^Effect size, Hedges’ g, based on predicted mean differences.

^c^Analyzed with linear mixed models (LMM), adjusted for repeated measures and clustering effects of twin pairs.

ES, effect size; TR, term control group.

### Group differences in sub-dimensions of stress

The subscales of child- and parent-related stress, in which significant differences between the PI and the PC group emerged, are displayed in Table 4. For all differences (whether significant or not) less stress was reported in the PI group. At 6 months, outcomes on one single subscale (Attachment) showed a significant difference between the preterm groups (t(278) =2.9, *P* = .004, ES =0.56). Fathers reported a similar difference on this subscale at age 1 (t(256) =2.8, *P* = .006, ES =0.55). More positive feelings were reported by the PI group at age 1 on the PSI subscale ‘Reinforces parent’ by both mothers (t(405) =2.3, *P* = .02, ES =0.46) and fathers (t(328) =2.0, *P* = .05, ES =0.44). Mothers in the PC group reported more stress related to lack of competence at age 1 (t(220) =2.3, *P* = .02, ES =0.47) and this difference between preterm groups persisted until age 7, with the largest effect reported at age 5 (ES =0.67).

**Table 4:** **Significant differences between the preterm control (PC) and preterm intervention (PI) groups on Parenting Stress Index (PSI), subscales**

	Mothers report		Fathers report	
	Subdomains of child-related stress	Subdomains of parent-related stress	Subdomains of child-related stress	Subdomains of parent-related stress
6 months		Attachment^b^		
1 year	Reinforces parent^a^	Competence^a^	Reinforces parent^a^	Attachment^b^
		Attachment^a^	Mood^a^	
2 years	Distractibility^a^	Competence^b^	Distractibility^a^	
	Adaptability^a^	Attachment^a^	Mood^a^	
	Demandingness^b^	Spouse^a^		
	Mood^b^			
	Acceptability^b^			
3 years	Distractibility^a^	Competence^c^	Distractibility^b^	
	Adaptability^c^	Attachment^a^	Mood^a^	
	Demandingness^a^			
	Mood^b^			
	Acceptability^a^			
5 years	Distractibility^c^	Competence^c^	Distractibility^c^	Spouse^a^
	Adaptability^c^	Attachment^b^	Adaptability^a^	
	Demandingness^b^		Reinforces parent^a^	
	Mood^c^		Demandingness^a^	
	Acceptability^b^		Mood^b^	
7 years	Distractibility^b^	Competence^b^		
	Adaptability^a^			
	Demandingness^a^			
	Mood^b^			
	Acceptability^b^			

All analyses generated with liners mixed models (LMM), adjusted for repeated measures and the clustering effect of twin pairs. a = *P* <0.05, b = *P* <0.01, c = *P* <0.001.

A pattern of PI parents perceiving their child as being happier than did PC parents emerged in fathers’ reports at age 1 (t(364) =2.2, *P* = .03, ES =0.45) and in mothers’ reports at age 2 (t(443) =2.6, *P* = .01, ES =0.51). This difference continued to be reported by fathers until age 5 and by mothers from age 2 until 7 with increasing ES, reaching 0.60 at age 7. Mothers in the PI group also reported less distractibility/hyperactivity, better adaptability to everyday challenges and a higher acceptability, indicating that infants in the PI group matched their parents’ expectations in a more appropriate way than those in the PC group. At age 5, both parents reported these differences (Table 4). Lastly, a significant difference in the subscale Spouse (t(235) =2.0, *P* = .05, ES =0.37), as reported by fathers, emerged at age 5 between the preterm groups. Analyses of the questions in this subscale indicated that fathers in the PC group spent less time with their partners than those in the PI group.

### Parental agreement concerning stress in the two preterm groups

The level of agreement between mothers and fathers were computed separately for the PI and the PC groups. Where significant differences in agreement between groups occurred, intraclass correlations in the PC and PI groups are reported and supplemented with *P*-values [29]. At age 2: Child Domain (ICC_PC_ =0.25; ICC_PI_ =0.69; *P* < .001), Parent Domain (ICC_PC_ =0.31; ICC_PI_ =0.64; *P* = .01), Total Stress (ICC_PC_ =0.24; ICC_PI_ =0.71; *P* < .001); age 3: Parent Domain (ICC_PC_ =0.26; ICC_PI_ =0.59; *P* = .01); age 7: Total Stress (ICC_PC_ =0.43; ICC_PC_ =0.65; *P* = .05) and age 9: DC (ICC_PC_ =0.36; ICC_PI_ =0.61; *P* = .04), Parent-Child Difficult Interaction (ICC_PC_ =0.20; ICC_PI_ =0.65; *P* < .001). Similar tendencies were reported on all other main outcomes except Child Domain at ages three and five, where agreement was at the same level.

### Did parents of preterm infants report more stress than parents of terms?

Stress reported by the PI and PC groups was compared with reports from the TR group in separate longitudinal and cross-sectional analyses (Figure 2).

### The PC and the TR groups compared

Group by age interactions were found in total and child-related stress as reported by mothers and in child-related stress as reported by fathers (Table 5). These three interactions are characterized by similar trajectories, as the TR group reported decreasing levels of stress from age one whereas the PC group reported stress at a higher and stable level across pre-school ages. Similar differences were found in several sub-dimensions (Table 5).

**Table 5:** **Significant interactions with age between the term reference (TR) group and the preterm groups**

Group by age interactions:	PSI dimension (mother or father)	F (df1, df2)	***P***
TR - and the PC group	Total Stress (Mo)	8.0 (1,641)	.005
	Child Domain (Mo)	12.5 (1,650)	< .0005
	Child Domain (Fa)	4.3 (1, 443)	.038
	Distractibility/Hyperactivity (Mo)	9.7 (1,660)	.002
	Distractibility/Hyperactivity (Fa)	4.6 (1,446)	.033
	Adaptability (Mo)	11.1 (1,659)	.001
	Demandingness (Mo)	7.7 (1,658)	.006
	Mood (Mo)	5.8 (1,670)	.016
	Competence (Mo)	5.6 (1,663)	.019
	Acceptability (Fa)	4.0 (1, 453)	.045
TR - and the PI group	Distract/Hyperactivity (Fa)	7.3 (1,473)	.007
	Mood (Fa)	5.4 (1,489)	.020

Fa, reported by fathers; Mo, reported by mothers.

Cross-sectional comparisons between the PC and the TR groups revealed significant differences in all main stress domains from age two until nine as reported by mothers. PC fathers reported more child-related stress than TR fathers at all follow-ups from age of one until seven and more Total Stress at age seven and nine. Both mothers and fathers in the PC group reported more Parent-Child Dysfunctional Interactions at age 9 than the TR group (*P* < .01).

### The PI and TR groups compared

No differences were found in the longitudinal report of total, child- or parent-related stress. In the distractibility/hyperactivity and mood sub-scales, PI fathers reported lower means before school-age and higher means at age 7 compared with TR fathers (Table 5). No significant cross-sectional differences between the PI and the TR groups emerged in reports of parenting stress from age 6 months until 9 years.

## Discussion

This study evaluated whether a sensitizing, early intervention affected the development of parenting stress among mothers and fathers of prematurely born children until age nine. The overall results indicated that the intervention reduced maternal stress, but to a lesser degree affected paternal stress in the intervention group. Different longitudinal patterns between the preterm groups were reported by PI and PC mothers on dimensions addressing child characteristics. PI mothers perceived their children as displaying higher adaptability and happiness throughout childhood than did PC group mothers. In addition, stress in the PI and PC groups was reported at quantitatively different levels at different follow-ups. PI mothers reported less total and child-related stress at all ages while PI fathers reported a similar difference from PC fathers at age five. The intervention may also have heightened the parental agreement within families as a stronger association between mothers and fathers responses was repeatedly found in the PI group compared with the PC group. Finally, parents in the PI group reported similar levels of parenting stress to those of terms at all follow-ups, while longitudinal and cross-sectional differences between the PC and TR groups increased with age. Thus, our main hypothesis was supported, as parents in the PI group reported stress below the levels of the PC group throughout childhood, and in fact was comparable to parents of term-born children.

In answer to the first question, a stress-subduing effect was found in the PI group concerning maternal perception of child-related stress in such aspects as adaptability and mood. More stress reported in these aspects of parenting stress has in particular been associated with difficulties in the parent-child relationship [5, 6]. The intervention had a sustained focus on support of early parent-child relationships. Parents were asked to initiate and facilitate social interactions whenever the child seemed to be ‘available’ but also to be sensitive to the child’s signs of distress and need for ‘time-out’. This may have initiated better-timed transactional patterns between PI mothers and their infants compared to the PC group. At the first follow-up (6 months) mothers in the PC group more often reported their children as fussy and in a bad mood when they woke up than the PI mothers did. This difference had disappeared by age one. However, from age two onwards, mothers in the PC group reported increased stress related to their children’s mood and adaptability, while the PI group mothers reported diminishing levels of stress until age seven. These results were dependent on the mothers’ answers to several PSI questions, but were strongly influenced by one item throughout childhood: ‘I feel that my child is very moody and easily upset’. Accordingly, reports from mothers at age one and later show that the PC mothers perceive less happiness, fewer smiles and fewer emotional responses from their children than the PI mothers. It has already been shown that premature babies may be less successful in showing strong positive arousal responses (for example, smiles) than full-term infants [32]. This suggests that the intervention had an influence on maternal stress, in terms of how mothers perceive their child and on their emotional relationship. The following paragraphs briefly discuss possible underlying mechanisms.

Heightened levels of stress are supposed to negatively affect maternal responsiveness [33]. Laucht, Esser *et al.* [34] studied the impact of maternal responsiveness on behavioral development in premature children. They found that problems such as anxiety and depressive mood decreased with age in children with highly responsive mothers, but increased where less sensitive mothering behavior was observed. We might speculate whether the intervention enhanced the ability of PI mothers to acquire realistic expectations and a deeper understanding about their children’s cues and need for support. Olafsen *et al.* [35] found that mothers who had participated in the intervention reported a strong association between stress and their children’s regulatory competence at 6 months. This may be an early indication of a more sensitive and synchronous interactional parent-child style. They may have been better able to read their child’s cues and ‘do what it takes’ to help their child in its immature regulation efforts. Another interventional aspect which may have decreased parenting stress in the PI group is the incorporation of the initial ventilation session, which may have strengthened the parents’ feelings of security and helped to improve their self-confidence [18, 34]. The session may also have influenced these parents’ establishment of a more robust parental attachment, which has been described as a powerful antecedent of the quality of mothers’ sensitive behavior [14, 19, 34]. The importance of maternal attachment has been documented by Coppola, Cassibba *et al*. [36] in connection with mother’s sensitivity at age 3 months. This was particularly powerful in mother-infant dyads with prematurely born children.

Even though maternal perceptions of child-related stress throughout childhood created the most significant differences between the preterm groups, the first reported difference appeared in parent-related stress, on the subscale Attachment at 6 months. Giving birth to a preterm child has been described as having a negative impact on maternal attachment [17, 33, 37]. The prolonged stay in the hospital and the NICU environments disrupts the natural physiological contact between mother and child. Borghini *et al*. [38] found that only 20% of mothers of preterm infants had a secure attachment representation at children’s age of 6 months compared to 53% of mothers of terms. According to Abidin, the PSI subscale Attachment was designed to assess the intrinsic motivation of parents in their roles as mothers or fathers [6], and this concept appears to be closely related to the development of a caregiving system as described by Walsh [15]. PC mothers reported significantly higher stress scores than PI mothers on several questions at 6 months, for example, ‘it takes a long time for parents to develop close, warm feelings for their children’ and ‘sometimes my child does things that bother me just to be mean’. These statements illustrate that a difference in experienced closeness and understanding of the child may have emerged between the PI and the PC mothers as early as 6 months post-discharge, with an impact on parental perception of stress.

Evans *et al*. [33] found that experiential avoidance and prenatal expectations were important predictors of maternal attachment and responsiveness styles. They suggested that avoidance could be used as a coping mechanism among mothers who struggled to deal with the new situation, but also as a predictor of weaker maternal attachment and responsiveness. As already mentioned, premature children may more often be characterized by a more serious expression than full-term children [32]. This may be associated with reports of emotional instability, observed for example as changeable moods, as have been more frequently reported among preschool preterms children than terms [39]. We therefore think that both maternal attachment and the infants’ expressions of emotionality might have been positively altered by the intervention. The toddlers may have regulated their mother’s feelings by their degree of susceptibility. When mothers in the PI group, guided by their new understanding of their individual child, were able to initiate interactions and elicit positive emotional expressions from their child, it may have become easier for them to establish an emotional closeness to the child and reduce their experiences of stress.

Deater-Deckard [5] emphasized that parenting stress is experienced as negative feelings toward both oneself and the child. More PC mothers expressed such negative feelings in terms of fewer smiles and lack of positive responses from their infants at age one than PI mothers. This produced a significant difference on the subscale ‘Reinforces parents’. This may be due to different expectations between these groups of mothers, but could also be a sign of subdued expressions of happiness among infants in the PC group, possibly influenced by a weaker emotional closeness to their mothers in these early months of life.

The early differences between the preterm groups included a difference in maternal experience of competence at age one, and subsequently significant differences in both competence and all child-related dimensions from age two until seven. Parental education has been found to be one of several key components in early interventions for preterm infants [18] and the MITP offered PI parents plenty of practical information and insights. We wonder whether the gradually increasing differences in maternal reports of stress between preterm groups, and a perception of poorer mother-infant adjustment among PC mothers, could be related to transactional mechanisms affecting the establishment of early parent-child synchrony and parental support of their child’s regulation [40]. Feldman *et al*. [41] found that better synchrony in early parent-child interactions at age 3 months predicted higher self-regulation skills among the children at age two. This was particularly important for children who were perceived by their parents as being difficult to manage [41]. This makes sense, as the difference between groups in stress concerning adaptability, distractibility, demandingness and acceptability were most evident at ages three, five and seven. Hauser-Cram *et al.* [42] reported similar increases in child-related stress among parents of children identified with disabilities. They identified variations in children’s self-regulation skills and mother-infant interactional skills as critical components.

PI fathers’ reports of stress seemed to be less affected by the intervention than those of PI mothers. The fewer significant differences between PI and PC fathers may also be influenced by great variability in father’s interventional participation. Negative correlations between paternal stress and PI father’s participation rates were evident on several measurement occasions. At age nine, correlations between stress and participation were significant in paternal perception of the child, father-child interactions, and their overall reports of stress. Similar correlations between paternal stress and the intervention participation have previously been reported by Kaaresen *et al.* at age one [22]. This may indicate that the associations are effects of intervention, but they may also be influenced by other factors such as differences in fathers’ motivation, knowledge, and so on. Interestingly, the highest negative correlation between reported stress and fathers’ participation were related to participation in the four home visits (r = -0.34). If this is a unique intervention effect it highlights the importance of including home visits in early intervention programs, in line with a recently published review [43]. The inclusion of fathers may also have promoted a higher degree of shared perception of stress between PI parents compared with PC parents. Morgan *et al.* [7] argues that better agreement regarding roles and challenges would be likely to produce more similar levels of parenting stress within families. A stronger agreement in the PI group was evident, especially at ages two and nine. A further interpretation of these results is difficult, since until recently, fathers have not been taken into account as independent informants in studies of parenting stress and child developmental outcomes [7].

Finally, we compared parenting stress between the preterm groups and the term reference group. Parents in the PI group reported child- and parent-related stress similar as TR parents, while both longitudinal and cross-sectional differences between the PC and the TR group throughout childhood did emerge. Even though the meta-analysis by Schappin *et al*. suggested that parents of preterm children have become less exposed to increased parenting stress during the past few decades [2, 8], our findings cannot confirm that conclusion. On the other hand, the occurrence of increased parenting stress frequently reported by parents of prematurely born children seemed to be eliminated by this intervention.

### Strengths and limitations

A major strength of this study is the high participation rates that were maintained throughout the study period, reaching 85% among mothers and 72% among fathers across groups even at age nine. Although randomization generated a high degree of equality between preterm groups in aspects of birth, medical and socio-economic variables, PI mothers did have an average of one year more education than the PC mothers. Maternal education has previously been reported to be negatively correlated with parenting stress [44] but in the latest meta-analysis by Schappin *et al.* [8] maternal educational levels were not found to influence any aspect of parental stress. Nevertheless, all our analyses controlled for the difference of one year in mean maternal education. A limitation related to the construction of the study lies in the nature of self-reported questionnaires. Data collected by the PSI questionnaire are a result of parents’ subjective perception of stress on a specific day. An inclusion of biological parameters, such as the measurement of cortisol, may have safeguarded against faulty conclusions. Data may also be influenced by the way questions are asked in the two questionnaires. In the PSI-SF, questions are expressed more directly (more directly problem-orientated formulations), which may have amplified differences between respondents in their perceptions of greater or less stress.

### Clinical implications

We have previously reported interventional influences on the longitudinal trajectories and cross-sectional differences on children’s behavioral outcomes [25]. Parenting stress is known to be closely correlated with children’s behavioral development [45] and relationships between parenting stress and child behavior outcomes will be reported in papers to follow. This study demonstrates how an early child-centered and family-focused intervention may reduce parenting stress across childhood. This is a finding, not only concerning families taking care of prematurely born children but possibly also for other children and families at risk.

## Conclusions

As hypothesized, we conclude that this sensitizing intervention reduced maternal parenting stress and positively influenced mothers’ perceptions of their children’s adaptability and happiness. Different longitudinal patterns in child-related stress were reported by PI mothers than by PC mothers throughout childhood. In all PSI main dimensions, significantly higher levels were reported by PC mothers and fathers at every age until the age of five. Stronger correlations were found in parenting stress reported by parents in the PI group than the PC group, indicating more shared perceptions of their children after intervention.

Finally, both parents in the PI group reported parenting stress similar to the term reference group at all follow-ups, whereas differences between the PC and TR group increased with age throughout childhood.

## Abbreviations

CI: confidence interval
CRIB: Clinical Risk Index for Babies
ES: effect size
GA: gestational age
ICC: intraclass correlation
LMM: linear mixed models
LS: Life Stress
MITP: Mother-Infant Transaction Program
NICU: neonatal intensive care unit
PC group: preterm control group
PD: Parental Distress
PI group: preterm intervention group
PSI: Parenting Stress Index
PSI: DC, subscale in PSI-SF named Difficult Child
PSI-FF: Parenting Stress Index-Full Form
PSI: P-CDI, subscale in PSI-SF named Parent-Child Dysfunctional Interaction
PSI: PD, subscale in PSI-SF named Parental Distress
PSI-SF: Parenting Stress Index-Short Form
SNAP: Score for Neonatal Acute Physiology
TISP: Tromsø Intervention Study on Preterms
TR group: term control group
TSS: Total Stress Score.
